# Influence of socio-family variables on parental assessment of the pragmatic development of children under 4 years of age

**DOI:** 10.3389/fpsyg.2024.1369949

**Published:** 2024-05-30

**Authors:** Iria Botana, Manuel Peralbo

**Affiliations:** Developmental Psychology Laboratory, Department of Psichology, University of La Coruña, A Coruña, Spain

**Keywords:** early pragmatic development, parental expectations about developmental timetables, determinants of development, context assessment, speech therapy intervention

## Abstract

**Introduction:**

Interest in pragmatic development and its assessment has increased in recent years, not only because of the predictive value of pragmatic impairments as warning signs in the detection of multiple developmental disorders, but also because of the consideration that pragmatics has received in the field of mental disorders. Current contexts of child development assessment require pragmatic assessment instruments that accurately define profiles and take into account the immediate context in which they develop. Parents' knowledge of their children's abilities is supported by exhaustive observation over time of regularities in their behavior. But it is true that the way a caregiver interprets behavior is mediated by multiple variables. The aim of the present study, therefore, is to shed light on the possible influence of parental belief systems on the assessment of children's pragmatic development by analyzing the relationship between sociofamilial variables and the assessment of pragmatic competence.

**Method:**

A total of 215 educational centers across Spain participated in the study. The final sample was of 262 parents of boys and girls between 6 and 48 months of age. The parental questionnaire for the evaluation of pragmatic development, The Pragmatics Profile, in an adapted Spanish version, was applied along with a number of items for the evaluation of parental beliefs.

**Results:**

Analyses confirm the existence of an effect of child development conceptions and other socio-familial variables on the assessment of pragmatic development between 6 and 48 months of age. Furthermore, the results indicate that better scores on pragmatic development are associated with parents with higher socioeconomic and educational levels, greater number of children and more interactionist conceptions and realistic.

**Conclusion:**

The effect of parental conceptions on the evaluation of pragmatics points to the need to obtain convergent measures in an area as complex as that of communicative development in early childhood, especially taking into account that an evaluation which is neutral and free from context is not possible or indeed desirable. Pragmatic development must be evaluated within this contextual framework and should take into account each of the variables present therein. Hence the complementarity between parental reports and performance-based test.

## 1 Introduction

As Hoff et al. (2014) notes, pragmatics has to do with the earliest phase of communicative intentionality, which, together with form and effect, constitutes one of the three components of the speech act (Reese, 1978). Pragmatics as a focus of study can be addressed from a variety of different areas of knowledge, including speech therapy, neurology, psychology, linguistics and sociology. In clinical linguistics, three major components are identified here; enunciative pragmatics, interactive pragmatics, and textual pragmatics (Gallardo, 2007). Enunciative pragmatics involves the communicative intention of the speaker, while interactive pragmatics focuses on the role of the receiver; between these two lies textual pragmatics, which deals with the analysis of the utterance itself. With the aim of achieving an in-depth understanding of the sequence of development during the early years, Halliday (1975), from the perspective of the socio-semantic study of language development, proposed three phases to which a universal character can be attributed, and which are enmeshed in a structure based on the so-called Hallidayean pragmatic functions. Initially, pragmatic development is based on the instrumental, regulatory, interactional, personal heuristic and imaginative functions, these developing mainly between birth and 18 months, then to give way to pragmatic, mathematical and informative functions. From the age of 24 months a third phase begins, which consists of the mastery of the adult system itself, this characterized by a reconceptualization of the notion of function, understood in the first phase as “use of language,” but now a component of the grammar of the mother tongue system that allows the development of ideational, interpersonal and textual functions.

It is within the first 5 years of life that pragmatic developmental milestones emerge at a faster rate (Oller et al., 2012). Before 12 months of age, children acquire the basic mechanisms of nonverbal communication. They must react to the human voice and identify familiar voices, pay attention to the adult's face, laugh out loud, or show responses to adult conversation through resources such as slang, pointing, or the use of communicative gestures during turn-taking (Berko and Bernstein, 2024). Understanding the role of context for the attribution of meaning requires the development of intentional behavior (from primary to secondary intersubjectivity), clearly identifiable at 9 months of age. At this stage, they actively participate in episodes involving joint attention (looking, attention-getting, etc.). Between 12 and 24 months of age, they already use language to make requests, express wishes or refusals, name objects, or share situations. From 24 months of age, they can ask contingent questions (Owens, 2011), inquire about the name and the reason for things, and begin to relate personal events. The symbolic function will make our knowledge about the meaning of things in the physical, psychological, and social world more accessible, allowing us to understand and give meaning to present and future situations, as well as plan actions based on this experiential knowledge. At the same time, they begin to tell fictitious stories (Sutton-Smith, 1986), paving the way for the more typical pragmatic developmental milestones of 36 months, which will consolidate the use of language. It is at this age when children accompany play with language, creating more complex narratives, and organizing their speech to make descriptions or recount stories heard (Pérez and Salmerón, 2006). Understanding the social rules that regulate interpersonal communication will allow them to better adapt to the context in their social exchanges (Messener, 1994). By 48 months, children can already adopt a variety of registers, especially in play situations (Owens, 2011), and these registers will provide training for refining narrative skills and adapting to the context needed in later stages. All milestones should follow the expected chronology to ensure harmonious pragmatic development.

Interest in pragmatic development and its assessment has increased in recent years, not only because of the evident predictive value of pragmatic alterations as the main warning signs in the detection of multiple developmental disorders (autism spectrum disorders, attachment bond disorders, attention disorders, etc.) but also because of the special consideration that pragmatics has received in the field of mental disorders. Proof of this is the recognition and categorization, for the first time, of pragmatic deficits in the diagnostic guidelines of the American Psychiatric Association (2013), (pragmatic) Social Communication Disorder (SCD) (González et al., 2015). Thus, alterations of a pragmatic nature that did not fit the clinical profiles established up to that point in time are now identified and recognized. For the first years of life, when the most significant pragmatic acquisition occurs (Santana et al., 2015), knowledge of the periods of pragmatic development is essential, not only for clinicians, but also for the family, the main context for stimulating communicative and social development.

For all these reasons, the current contexts of child development assessment require instruments of pragmatic evaluation that define profiles precisely and take into account the immediate context in which they are developed. Despite this, the number of methodological studies directly related to the evaluation of this in infants is limited (Portilla and Mogollón, 2015). The study by Prieto et al. (2021) includes an exhaustive analysis of the instruments available in Spanish. It highlights the difficulty of creating communicative situations with ecological validity in the clinical context, thus underlining the usefulness of parental reports as a complement to assessment, despite their limitations and possible biases (Jackson-Maldonado et al., 2013). Also, Šmit Brleković and Kuvač Kraljević (2023) confirm the usefulness of parental reports in the assessment of early language development, since their multidimensional nature, especially considering the limitations of other methodologies, make them a valuable source of information in the early stages of communicative and linguistic development. This is especially relevant in the assessment of pragmatic development before the age of 4, when evaluations of direct performance are difficult, and indeed at times impossible. This barrier is of particular relevance in the clinical setting, where such difficulties compound those arising from possible disorders or alterations in neurodevelopment.

The creation of parental questionnaires has made it possible to overcome these difficulties with a high degree of reliability, not only in the field of communication and language, but in development generally (Gonzalez and De Pedro, 2023). Despite their application and usefulness, some of their limitations can be pointed out. These include social desirability bias, that is, bias due to the experimenter, bias attributable to demand characteristics, and the difficulty that some questionnaires have in obtaining accurate information about complex behaviors (Anis et al., 2020).

These and other biases can influence the way that parents perceive and evaluate their children's development. Undoubtedly, the knowledge that parents have about their own children's skills is undoubtedly supported by 56 prolonged and daily observation of their behavioral regularities (Guiberson et al., 2011), but they are also influenced by the knowledge coming from their socio-cultural group, forming true implicit theories about development and education. What goals should be achieved, when, through what tasks and actions, and that role children and social agents have in this process, are part of the representations we build as part of a society and are determinants of our actions and ways of interacting. The ideas that parents have about their children and about themselves as parents influence their actions (Triana, 1991; Sigel et al., 2014). This everyday knowledge, which is not in itself accurate, but rather functional, allows parents to be considered as reliable informants about their children's cognitive, motor, social, and also communicative skills, and this is of particular importance for the assessment of pragmatics at an early age (Šmit Brleković and Kuvač Kraljević, 2023). Its reliability has been shown in studies by Suskind et al. (2018), Andrés-Roqueta et al. (2021), and Botana (2021), for example. In these, significant correlations were found between the results of parental questionnaires and those from clinical evaluation by means of direct tests. Specifically, in the study by Botana (2021), a very significant convergence is observed in the assessment of pragmatic development up to the age of three, with divergences then being seen in the year-four group for both measurement instruments. This discrepancy coincides with the variation found in the solidity and strength of parental ideas on the determinants of development at the beginning of formal schooling, around 36 months, in the study by Ribao et al. (2021). On the other hand, at the age of three, there are notable modifications in structural pragmatic aspects due to the role of verbal language in the assessment of pragmatics. The possibility that parents might be biased in the information they provide when answering a questionnaire should itself be a parallel object of study in the evaluation of pragmatic child development. How an adult interprets children's behaviors has to do fundamentally with three aspects: the characteristics of the children, the characteristics of the parents, and the characteristics of the context, all of which contribute to generating expectations and attributions about children's behavior and its causes (Mills and Rubin, 1990).

The parental belief system is a complex construct. It involves shared beliefs, guided by cultural values about the goals of child development and the socializing practices that lead to the achievement of that development. Such evolutionary-educational ideas (Palacios and Rodrigo, 1998; Hidalgo and Hidalgo, 2003; Greenfield and Keller, 2004) are reflected in parents' conceptions of the determinants of development, about the attribution of the causes of behavior and development (to heredity, the environment, or the interaction between the two), and is related to parents' conceptions of what parenting itself entails. In the most innatist conceptions, development is solely determined by genetic factors. The most environmentalist perspectives attribute maximum competence in development to the environment. Meanwhile, interactionists position themselves somewhere in the middle, recognizng both the influences of the context and those of a genetic basis, and adopting a combination of beliefs in the causal attribution of child development. In addition, knowledge about the developmental calendar, the skills that the caregiver believes are developed at the stage in which the child is currently at, will influence the interpretation of their actions. The presence or absence of a behavior can be valued differently depending on parental expectations as to its probable age of acquisition. These expectations explain at what moment parents expose their children to tasks and propose goals for the achievement of which they consider parental support more or less necessary. Without doubt, the origins of expectations about the developmental milestones is cultural and takes form differently in each cultural group or subgroup, but it is also true that expected behaviors condition the beginning of parent-child interaction and its ongoing nature (Siegel et al., 1992; Palacios and Rodrigo, 1998; Hidalgo and Hidalgo, 2003).

In the case of notions about the determinants of development (the nature-nurture controversy) it seems clear that these mediate the behavior of caregivers insofar as they position them as more or less active agents of child development. What does not depend on these notions does not have to give rise to setting specific goals to achieve them. In the same way that if everything depends on these notions, the expectations generated about the effect of their educational activity will very often lead to frustration. Likewise, expecting developmental progress too soon can lead to forcing children into activities for which they are not ready, with a negative effect on the development of their self-efficacy or sense of personal competence. Dysfunctional expectations are related to action plans and consequences that are not positive for child development, and that condition the way in which children and caregivers interact. Excessive optimism about when skills can be achieved, as well as dysfunctional pessimism, can have consequences for the subjective assessment of pragmatic development. Expectations about the developmental milestones and the determinants of development are aspects that mediate the way in which parents and children interact within the area of proximal development (Bruner, 1983; Palacios, 1987).

In the case of pragmatic development, when parents or caregivers report on their children's pragmatic development, they generally refer to their communicative effectiveness. This usually includes an experience-based assessment of four pragmatic indicators that regulate the basic communicative power of children's interaction (Dewart and Summers, 1995): (a) communicative functions; (b) response to communication; (c) interaction and conversation; and (d) verbal variation according to context. These indicators are included in the TPP(e) (Botana and Peralbo, 2022).

These four parameters are decisive for estimating the acquisition of pragmatic skills in the early stages of development, beyond other more structural or formal requirements, such as morphosyntactic ones (Fernández, 2019).

The literature supports the effect of social, family and cultural variables on various aspects of child development (Kluczniok et al., 2013). It provides evidence of the relationship between coming from lower socioeconomic status (SES) environments and the increased risk of language delay (especially its lexical and morphosyntactic components), as noted by Cohen et al. (2020). Similarly, parents' investment in their children varies with socioeconomic status, and socioeconomic status itself appears to be related to vocabulary development and to a greater presence of child-directed speech in early childhood (Rowe, 2008).

For this reason, and since parental reports are one of the most common assessment instruments in the evaluation of language development, it seems appropriate to determine the extent to which their reliability also extends to the assessment of pragmatic development. To this end, the following objectives have been established:

- To analyse the influence of the educational level of the family on the results of the TPP(e).
- To analyse the influence the socioeconomic status of the family on the results of the TPP(e).
- To see whether the number of siblings leads to any differences in the results of the TPP(e).
- To analyse differences in the results of the TPP(e) due to parents' conceptions of the determinants of development.
- To see if there are any differences between parents' conceptions of the determinants of development and their level of knowledge about the evolutionary calendar.
- To analyse the relationship between this knowledge of the evolutionary calendar and the results of the TPP(e).

## 2 Method

An ex post facto study of a fundamentally quantitative, descriptive and correlational nature was conducted. As is characteristic of this type of design, there is no intentional manipulation of and independent variable, participants have the characteristics required by the research, and consequently there is no possibility of controlling the variables and their effects, which have already occurred previously. In this type of design, internal validity is lower than external validity, because although extraneous variables cannot be controlled, they deal with more natural and representative situations (Shaughnessy et al., 2007).

### 2.1 Participants

First, a selection of pre-elementary schools across all the Spanish autonomous regions was made. For this purpose, a total of schools were identified, all of them either public and private centers of the first to the fourth years of primary education. From there, a distribution was made based on the percentage of the Spanish population in each autonomous region, resulting in the distribution as detailed in Table 1.

**Table 1:** Distribution of the selection of schools.

**Autonomous region**	**Population**	**Percentage of population**	**Schools invited**	**Schools participating**
Andalucía	8,500,187	17.87%	75	44
Cataluña	7,792,611	16.38%	68	41
Comunidad de Madrid	6,750,336	14.24%	60	9
Comunidad Valenciana	5,097,967	10.67%	45	18
Galicia	2,690,464	5.68%	25	24
Castilla y León	2,372,640	5.02%	21	7
País Vasco	2,208,174	4.67%	20	17
Canarias	2,177,701	4.58%	19	6
Castilla-La Mancha	2,053,328	4.32%	18	6
Región de Murcia	1,531,878	3.20%	13	8
Aragón	1,326,315	2.79%	12	3
Islas Baleares	1,176,659	2.47%	10	6
Extremadura	1,054,776	2.23%	9	7
Principado de Asturias	1,004,686	2.14%	9	9
Navarra	664,117	1.39%	6	4
Cantabria	585,402	1.23%	5	5
La Rioja	319,892	0.67%	3	0
Melilla	85,170	0.18%	1	0
Ceuta	83,117	0.18%	1	1
España	47,475,420	100%	420	215

Of the 420 schools which were sent the information by means of email and were invited to participate, 215 accepted and informed the families of their children about this. Finally, having received the information through the school in question, 271 parents of boys and girls between 6 and 48 months age participated in the study. Participants (*N* = 262) had a mean age of 28.45 months (*SD* = 10.449). The data on asymmetry (−0.336) indicate a slightly skewed age distribution above the mean. Kurtosis data (−0.701) indicate a platykurtic curve with lighter tails than expected under normal distribution. The sample presents a mean of 1.01 siblings (*SD* = 0.793), with asymmetry (0.219), which shows more values above the mean than below, and a kurtosis (−0.819) that, as in the case of age, indicates a platykurtic distribution with lighter tails than expected in a normal distribution.

**Figure 1:**
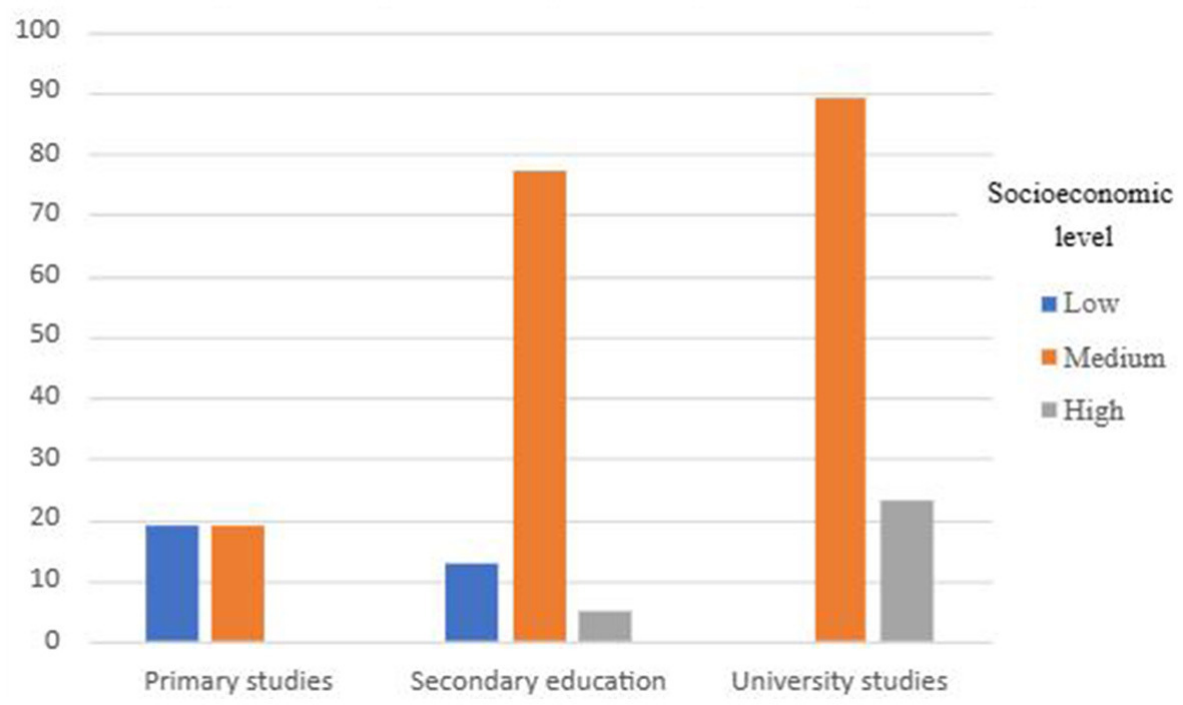
Distribution of the sample according to socioeconomic level and educational level.

As can be seen in Figure 1, there is a greater representation of the medium socioeconomic level and the medium and high educational levels, compared to the others. Analysis of the relationship between these variables shows a significant association between them $\chi_{\left(4,245\right)}^{2}$ = 730,879, *p* < 0.001.

Regarding the exclusion criteria, cases in which the parents reported alterations in development were excluded through a form in which they were asked for information about whether their child presented any of the following difficulties: moderate or severe hearing loss, severe visual impairment, syndromes of a genetic origin, neurodevelopmental disorders, prematurity, central nervous system disorders, long periods of hospitalization and/or institutionalization. Based on these criteria, from the initial collection of participants, a total of 9 were discarded, without any analysis of their characteristics to reveal indications that might justify their consideration as a subsample. The final sample was 262 fathers and mothers.

### 2.2 Variables and measuring instruments

#### 2.2.1 Pragmatic competence

The TPP(e) questionnaire was used, as adapted by Botana and Peralbo (2022), and based on an original interview by Dewart and Summers (1995). The analyses of the adapted Spanish version show a Cronbach's alpha of 0.976 and a McDougal's Omega of 0.98, values that can be considered very adequate. The TPP(e) evaluates early pragmatic development through 35 items with three response options grouped into three axes of pragmatic development:

Axis A: communicative functions, including how a child can express intentions, such as requesting, greeting and giving information, through communicative behaviors.
Axis B: communicative response, indicating the way in which a boy or girl reacts and understands the communication of other people.
Axis C: interaction and conversation, classifying the way in which children participate in conversation, as a part of social interactions relating to initiation, turn-taking, repair, etc.

It provides a direct score and a centile score for each axis as well as a total score.

#### 2.2.2 Conceptions about the determinants of development

Palacios and Rodrigo (1998) refer to these, noting the problem of the relationship between heredity and the environment in determining human development. Thus, for example, innatist parents would be those who attribute the causes of their five children's development to their genetic characteristics. For interactionists, the main determinant of children's development is the relationship between environmental and genetic factors Environmentalists, meanwhile, consider the influence of the environment as the main cause of development. To evaluate these, three questions from the original study mentioned above were used: (1) Why do you think some children are more intelligent than others? (2) If a 4 or 5-year-old child is very shy, do you think that their parents can do something to make them less shy? and (3) Sometimes you would like your child to be able to do something that seems important to you. In those cases, you... Responses were coded as 1 (innatist responses), 2 (interactionist responses), and 3 (environmentalist responses).

#### 2.2.3 Expectations regarding the developmental milestones

These refer to the age at which parents believe that their children will achieve certain normative developmental achievements in childhood (Hidalgo and Hidalgo, 2003). In this study they have been classified as:

Pessimists: parents assume that developmental goals are achieved at an older age than the age at which this should really happen.
Realistic: parents show knowledge appropriate for the normative age of acquisition.
Optimists: parents believe that developmental achievements are achieved earlier, that is, prior to what is actually normative.

Inspired by the study by Palacios (1987), six questions were developed that elicit the knowledge or beliefs of the caregiver regarding the moment at which it is expected that children will reach a milestone; for this, certain milestones of child development were selected and three answer options were associated with them. (1) Next, I am going to ask you a series of questions on what you think about children's development. Please indicate the option that is closest to what you think. At what age do you think children sit up without support? (2) At what age do you think children begin to take their first steps? (3) At what age do you think they can control their own pee and poop? (4) At what age do you think they start to say their first words? (5) At what age do you think they start pointing out what they want or what catches their attention? (6) At what age do you think they start saying their first two-word sentences (e.g., *mamá-teté*or*papá-teté)*. Answers which were incorrect because they cited times that were too early were coded as 1; correct answers were coded as 2; and incorrect answers because they were too late were scored as 3.

#### 2.2.4 Socioeconomic level

Information on the educational level of the parents was collected directly through the questionnaire sent to the parents. To this end, parents were asked to state their socioeconomic level from three possible options: “low level” (1), “medium level” (2) and “high level” (3). The family's economic level is related to the type of material resources and experiences that can be achieved in the social context in which families develop.

#### 2.2.5 Educational level

This variable was used to collect information on the educational level of the parent who completed the questionnaire, with the answer options “incomplete or primary studies” (1), “secondary education: vocational training or equivalent” (2) and “higher or university studies” (3). Educational attainment is one of the sources of information that contribute to the formation of parental beliefs.

#### 2.2.6 Number of siblings

The number of siblings is a relevant variable due to its relationship with the developmental milestones, since it is one of the sources of information on what is expected or not in child behavior. Thus, information on the total number of siblings was collected through a direct question in which the parents provided the total number of siblings of the child being evaluated.

### 2.3 Procedure

Once the sample was selected, information including the objectives and procedures was sent to the schools for parental participation, indicating that the one with the higher educational level should answer the questionnaire. In addition, informed consent was acquired, and other recommendations of the ethics committee were complied. From the schools the information was sent to the families, and by means of a link to Google Forms they were able to access the questionnaire, which contained the 35 questions of the TPP(e), six questions on the developmental milestones, and three questions on the determinants of development. On this form, one item could be seen on each screen, so that it was necessary to answer a question before moving on to the next. The items corresponded to the TPP(e) questionnaire and to those used to classify parental conceptions about the developmental milestones and the determinants of development.

A 3-week deadline for receiving responses was set. From this moment, the data received was recorded and processed.

### 2.4 Data analysis

Statistical analyses were performed using SPSS (v.29). Missing values were considered to be those cases in which the response to any of the variables under study was omitted.

In order to have a measure that would allow us to group parents according to their conceptions of the determinants of development, the median was calculated for the set of responses. Given that the three questions on determinants of development had answer options ordered from 1 to 3 (innatist = 1, interactionist = 2 and environmentalist = 3), and since each parent could score differently on each item, it was considered that the median, as a measure of the central tendency, would be the statistic that best reflected the parent's perspective. In the case of expectations about the evolutionary calendar, the following were taken into account: the number of correct answers, the number of errors by default (excessively optimistic), the number of errors in excess (too pessimistic) and the total number of errors. In this way, it was possible to assess the level of knowledge that each informant possessed about normative development.

The data were tested for normal distribution using the Kolmogorov–Smirnov test and for homogeneity of variances using the Levene test.

Differences between groups were tested using one-way ANOVA, followed by the Games-Howell *post-hoc* test, which is appropriate when there are groups with different numbers of subjects, as in this case, and equality of variances is not assumed. Finally, a correlational analysis was performed using Spearman's rho.

## 3 Results

### 3.1 Descriptive analysis

First, parents were classified according to their conceptions of developmental determinants and developmental milestones. In the first case, of the 262 participants, 87.5% were interactionists, followed by 6.8% environmentalists, and 5.7% innatists. In the case of the evolutionary calendar, the most frequent trend was classified as “realist” (83.2%), followed by “dysfunctional pessimism” (13%) and, finally, with a clearly lower representation, “dysfunctional optimism” (3.8%).

We then tested whether the data obtained from the TPP(e) conformed to a normal distribution. The results of the Kolmogorov–Smirnov test are highly significant in all the axes of the TPP(e) and in the overall result of the questionnaire, so the null hypothesis that the data respond to a normal distribution is rejected. The A-axis scores show a distribution of *Z*_(262)_ = 0.213, *p* < 0.01, with an asymmetry of −0.602 and a kurtosis of −1.068. The B-axis scores show a distribution of *Z*_(262)_ = 0.143, *p* < 0.01 with an asymmetry of −0.426 and a kurtosis of −0.872. On the C-axis, the distribution obtained is *Z*_(262)_ = 0.105, *p* < 0.01 with an asymmetry of −0.565 and a kurtosis of −0.566, and in the total score it is *Z*_(262)_ = 0.159, *p* < 0.01 with an asymmetry of −0.548 and a kurtosis of −0.919. Overall, a negative skewness together with a negative kurtosis suggests that the distribution is skewed to the left and has shorter and less heavy tails than a normal distribution. This indicates that most of the data are concentrated near the mean, and there are relatively few outliers compared to a normal distribution.

### 3.2 Analysis of variance and *post-hoc* tests

#### 3.2.1 Influence of the family's educational level on the results of the TPP(e)

The results of the ANOVA showed significant differences in the score on the B-axis, and the response to the communication of the TPP(e) between parents with primary education (*M* = 19.66; *SD* = 5.095; *F*_(2, 244)_ = 7.377; *p* < 0.015; η^2^ = 0.0.57), secondary studies (*M* = 21.41; *SD* = 5.050; *F*_(2, 244)_ = 7.377; *p* < 0.015; η^2^ = 0.0.57) and higher studies (*M* = 23.10; *SD* = 5.010; *F*_(2, 244)_ = 7.377; *p* < 0.015; η^2^ = 0.0.57).

*Post hoc* contrasts only show significant differences on the B-axis between groups 1 and 3 (*Diff* = −3.440; *p* = 0.002), 2 and 3 (*Diff* = −1.688; *p* = 0.045). In both cases, group 3 (high educational level) has the highest scores.

#### 3.2.2 Influence of the family's socioeconomic status on the results of the TPP(e)

The results of the ANOVA again showed significant results in the score obtained on the B-axis of the TPP(e) according to the socioeconomic level reported by the family: parents with low socioeconomic status [*M* = 19.28; *SD* = 4.595; *F*_(2, 244)_ = 4.919; *p* = 0.008; η^2^ = 0.0.92], medium [*M* = 22.29; *SD* = 5.186; *F*_(2, 244)_ = 4.919; *p* = 0.008; η^2^ = 0.0.92] and high [*M* = 22.39; *SD* = 4.947; *F*_(2, 244)_ = 4.919; *p* = 0.008; η^2^ = 0.0.92].

In *post hoc* analyses, as in the case of level of education, differences in socioeconomic level are also concentrated on the B-axis, between groups 1–2 (*Diff* = −3.011; *p* = 0.039), and 1–3 (*Diff* = −3.112; *p* = 0.004). In all cases, the differences are in favor of group 3 (high socioeconomic level).

#### 3.2.3 Differences in TPP(e) results due to the number of siblings

Next, an ANOVA was performed using the number of siblings as an independent variable (categorically coded as 0, 1, 2, and 3), with the three dimensions of the TPP(e) and the total score in the parental questionnaire as dependent variables. In this analysis no significant differences were found between the means of the four established groups.

However, a posteriori contrasts carried out with the Games Howell test show differences on the A-axis, between 0 and 3 siblings (*Diff* = −6.389; *p* < 0.001), between 1 and 3 siblings (*Diff* = −4.675; *p* = 0.005) and between 2 and 3 siblings (*Diff* = −4.970; *p* = 0.012), in all cases favorable to those families with a greater number of siblings. On the B-axis, the differences only appear between 0 and 3 siblings (*Diff* = −1.736; *p* = 0.014). On the C-axis, there are no significant differences. Finally, the results also show differences in the total score of the TPP(e) between 0 and 3 siblings (*Diff* = −9.639; *p* = 0.002).

#### 3.2.4 Differences in TPP(e) results due to conceptions about the determinants of development

An ANOVA was conducted in order to see whether there were significant differences between the responses to the TPP(e) and the conceptions of the determinants of development (coded as 1, 2, and 3, as indicated). In the analyses conducted, the groups of parents showed differences in the total score for the TPP(e) depending on their conceptions of the determinants of development. The three groups of parents, innatists (*M* = 54.93; *SD* = 20.03), interactionists (*M* = 74.25; *SD* = 21.97) and environmentalists (*M* = 64.72; *SD* = 24.70) achieved differing overall scores. Analyzing the data by axes, it can be seen that differences exist between axes B and C. In the case of axis B, differences are observed between environmentalists [*M* = 20.33; *SD* = 4.70; *F*_(2, 261)_ = 7.534; *p* < 0.001; η^2^ = 0.59], interactionists [*M* = 22.37; *SD* = 5.10; *F*_(2, 261)_ = 7.534; *p* < 0.001; η^2^ = 0.59] and innatists [*M* = 17.53; *SD* = 3.48; *F*_(2, 261)_ = 7.534; *p* < 0.001; η^2^ = 0.59]. In the case of the C-axis, differences were found between envirtonmentalists [*M* = *13.56; SD* = 6.87; *F*_(2, 261)_ = 6.757; *p* < 0.001; η^2^ = 0.53], interactionists [*M* = 16.44; *SD* = 5.46; *F*_(2, 261)_ = 6.757; *p* < 0.001; η^2^ = 0.53] and innatists [*M* = 11.73; *SD* = 5.56; *F*_(2, 261)_ = 6.757; *p* < 0.001; η^2^ = 0.53]. In all cases, parents with more interactionist conceptions obtain a higher average score.

Next, in the *post hoc* analyses, carried out with the Games Howell test, suitable in cases where the groups do not have equal variances, it can be seen that parents with more interactionist conceptions had higher mean scores on all axes of the PPT(e), A-axis (*Diff* = 9.772; *p* = 0.018), B-axis (*Diff* = 4.835; *p* = <0.001), C-axis (*Diff* = 4.705; *p* = 0.015), as well as in the total score here (*Diff* = 19.312; *p* = 0.006) than the group of parents with more innatist conceptions, followed by the group of parents with environmentalist conceptions.

#### 3.2.5 Differences between parents' conceptions of the determinants of development and the level of their knowledge about the evolutionary calendar

An ANOVA was carried out using the type of conception of the determinants of development (environmentalists, interractionists, innatists) as a factor, and as dependent variables the number of correct answers in the evolutionary calendar questionnaire, the number of errors by default (dysfunctional optimism), excess (dysfunctional pessimism), and the total number of errors. The results show significant differences between the three types of determinants in number of correct answers [*F*_(2, 259)_ = 39.59; *p* < 0.001, η^2^ = 0.234], in the number of errors by default [*F*_(2, 259)_ = 28.16; *p* < 0.001; η^2^ = 0.179], the number of errors due to excess [*F*_(2, 259)_ = 35.435; *p* < 0.001; η^2^ = 0.215], and the total number of errors [*F*_(2, 259)_ = 38.48; *p* < 0.001; η^2^ = 0.229].

In the *post hoc* comparisons, carried out with the Games-Howell test, it is clear that parents with more interactionist conceptions about the determinants of development are the ones who provide a greater number of correct answers in the questions on the evolutionary calendar, significantly above environmentalists (*Diff* = −3.84; *p* < 0.05) and innatists (*Diff* = 5.00; *p* < 0.05), with no differences between these. In other words, they have more realistic conceptions about the evolutionary moment at which the achievement of a milestone should be expected. On the other hand, those with the most errors by default (due to optimism) were those who had more environmentalist conceptions (*Diff* = 1.880 with interactionists, and *Diff* = 2.178 with innatists, *p* < 0.05 in both cases), with no differences in this type of error between interactionists and innatists. In the case of errors due to excess (pessimism), it is the innatist parents who differ significantly from both the interactionists (*Diff* = 8.395; *p* < 0.05) and the environmentalists (*Diff* = 8.267; *p* < 0.05), with no differences between environmentalists and interactionists for this type of error. Finally, considering the sum total of errors (whether due to over-optimism or over-pessimism) differences were found between the three types of parents. Among environmentalists and interactionists (*Diff* = 2.008), environmentalists and innatists (*Diff* = −6.089), interactionists and innatists (*Diff* = −8.097). In all cases, it is the interactionist group that makes the fewest errors (all differences were significant at 0.05), followed by the environmentalist group, and with the innatist group having the highest score here. That is, parents who are pessimistic in terms of the evolutionary calendar are essentially innatists. The dysfunctional optimists are essentially environmentalists.

#### 3.2.6 The relationship between knowledge about developmental milestones and the results of the TPP(e)

A Spearman correlation analysis was carried out to explore the relationship between the conceptions of the determinants of development and the scores obtained in pragmatic development. Table 2 presents the resulting correlation matrix, which shows the correlations between the different conceptions and the axes of pragmatic development.

**Table 2:** Correlation matrix between the conceptions of the determinants of development and the scores obtained in pragmatic development (Spearman).

	**Realistic conceptions (*n* = 218)**	**Pesimisstic conceptions (*n* = 34)**	**Optimistic conceptions (*n* = 10)**
Axis A; Communicative functions	0.157	−0.169^*^	−0.038
Axis B; Response to communication	0.233^**^	−0.202^**^	−0.087
Axis C; Interaction and conversation	0.179^*^	−0.173^*^	−0.074
Total score of the TPP(e)	0.180^*^	−0.171^*^	−0.067

^*^p <0.05.

^**^p <0.01.

Significant correlations were observed between realistic conceptions and several axes of pragmatic development. Specifically, positive correlations were found with axis B, which addresses response to communication (ρ = 0.233, *p* < 0.001), as well as with axis C, which focuses on interaction and conversation (ρ = 0.179, *p* = 0.004). These findings suggest an association between realistic conceptions and greater development of communicative and interaction skills.

On the other hand, pessimistic conceptions showed significant negative correlations with all axes of pragmatic development. Moderate negative correlations were observed with axes A (ρ = −0.169, *p* = 0.006), B (ρ = −0.202, *p* < 0.001), and C (ρ = −0.173, *p* = 0.005). These results indicate an association between pessimistic conceptions and lower development in areas such as communicative functions, response to communication, and interaction and conversation.

Correlations between optimistic conceptions and axes of pragmatic development did not reach statistical significance.

Taken together, these findings suggest that conceptions of developmental determinants may be related to pragmatic development in early childhood, highlighting the importance of individuals' beliefs and perceptions in the process of developing.

## 4 Discussion

The analyses confirms the existence of an effect of the socio-family variables analyzed on pragmatic development between 6 and 48 months. These results show that it is parents with more interactionist conceptions on the determinants of development, with predominantly realistic conceptions about the developmental milestones, higher educational and socioeconomic level, and with more than one child, who obtain the highest scores on the pragmatic evaluation questionnaire TPP(e).

In relation to the objectives set out, the analyses show a significant correlation between the educational and socioeconomic level of parents and the scores obtained in pragmatic development, with the children of parents with a higher educational and socioeconomic level obtaining clearly better scores here. These results are in line with those reported in the study by Ajayi et al. (2017) from a sample of 1.580 children, which confirms the effect of socio-family variables such as educational level, socioeconomic level and nutrition on assessments of children's cognitive development. Also in the study by List et al. (2021), parents of higher socioeconomic status believed that their investment in their children influenced their children's development. Such investment in resources and experiences is, in fact, one of the decisive factors in the production of children's skills during the early stages of development. It has also been shown that this kind of investment differs according to socioeconomic status, as noted by Hoff (2003) and Rowe (2008).

Current studies in this area discuss the implications for understanding the possible effects of family structure on language development (Havron et al., 2022), pointing to possible effects of family structure on various aspects of language development. These findings do not allow us to establish a clear profile of the family structure that favors language development, with controversial issues regarding the positive effect of the number of older siblings, for example, in the study by Tsinivits and Unsworth (2021), vs. the negative effect of the number of older siblings in that by Havron et al. (2022). In the present study, we analyzed the relationship between the number of siblings, without specifying the order of these, and scores in pragmatic development. The experience of parenting with more than one child is undoubtedly a relevant source of information toward understand the extent of developmental determinants and toward a better understanding of typical developmental patterns (Guiberson et al., 2011). But sibling interaction is also a driving force for pragmatic development.

The importance of parents' experience and knowledge on development are also related to socioeconomic and educational status, as reported by Hutchinson and Wojcik (2022). In their research they find that many adults with various cultural and professional backgrounds are unsure about how children develop, and the authors note differences in the way parents and non-parents think about development. Even so, it is important to bear in mind that, in our sample, the variable analyzed was the number of siblings, without including the order of birth, which hence does not assess differences in terms of being older or younger children, which would really allow us to discriminate the impact of experience and early exposure to communicative formats with siblings more thoroughly.

On the other hand, our results allow us to affirm that the children of those parents whose conceptions on the determinants of development are more interactionist and have a greater knowledge of the evolutionary calendar do obtain better scores in pragmatic development. At the same time, a higher educational and socioeconomic level, and the presence of siblings, also have a positive effect on the scores obtained in the pragmatic competences of the minors here.

These findings are consistent with those found in other studies on the favorable effect of parental conceptions on the assessment of their children's linguistic or non-linguistic achievements. For example, mothers who believe that the environment can positively influence child developmental outcomes are known to initiate quality language use with their children, which in turn correlates with more advanced lexical and syntactic skills (Gamble et al., 2009; Sigel et al., 2014; Wang et al., 2022).

We know that the ideas parents have about their children and themselves as parents influence their actions (Triana, 1991; Sigel et al., 2014), and therefore it is not surprising that those parents with more interactionist ideas, who perceive a relationship between their actions and their children's achievements, are the ones who obtain the highest scores in the assessments of their children's pragmatic skills.

In addition, the results of the present study show a relationship between expectations as to the maturation calendar and the assessment of pragmatic development, parents with more realistic conceptions obtaining higher scores on the pragmatic development questionnaire. These results are in line with other studies in various related areas of child development and academic achievement. We know that the structural characteristics of the family and the educational beliefs of the parents are related to the quality of the language acquisition process, and that the quality of the process is directly related to the child's outcomes (Kluczniok et al., 2013). But this is certainty not enough. Current lines of research are rigorously addressing the effect of family and contextual variables in the study of specific skills, examples here being studies of executive functions in ASD (Quero and Cañete, 2022) and in the assessment of academic performance (Rodríguez-Santero and Gil-Flores, 2018; Gonzalez and De Pedro, 2023), level of vocabulary (Cohen et al., 2020) and motor development (Jiménez et al., 2020). Again, these findings should not be surprising, since knowing the evolutionary moment at which a milestone should appear will undoubtedly favor the process by which parents, especially the most interactionist ones, propose appropriate spaces and contexts so that this milestone can occur, thus acting as facilitating agents of development.

Parents' expectations as to the developmental milestones, their causal attributions, and their position on the nature-nurture continuum, as well as their educational and socioeconomic status, will mediate not only the assessment of achievements, but also the way in which they adjust the stimulation of pragmatic milestones in the early years. A caregiver whose beliefs place them as an agent of their child's development will directly and indirectly contribute differentiated strategies to the interactions they share in daily life (Hidalgo and Hidalgo, 2003).

In the field of early care, where pragmatic development is of crucial diagnostic importance (Trivette et al., 2010; McWilliam, 2016), such evidence has been used as an anchor for family-centered practices, thus establishing routines as the main format for study and intervention. The family continues to be seen as the main context in which the most significant development opportunities of the early years take place, as it had been seen in earlier ecological theories (Bronfenbrenner, 1986). Knowing to what extent the opportunities for communicative development—the formats defined by Bruner (1982)—are mediated by conceptions of development in parents and their social contexts, and to what extent they shape the zone of proximal development (Vigotsky, 1934; Vygotsky, 1984) in which children make their communicative advances, is important from the point of view of speech therapy. Not only because of the interest in knowing about parental perceptions and their conditioning factors to understand how these influence child development, but also toward having resources that allow them to be modified, since these are not static but change according to parents' own experiences as parents (Hidalgo and Hidalgo, 2003). Speech therapy evaluation requires new tools, and intervention here also echoes these shortcomings. In 2016, as a result of the work by Escorcia et al. (2016), it was pointed out that there was a need to reflect on the transformation of speech therapy services and how this change should be assumed by practice here. Subsequent research on evidence-based practices (Strain, 2018) suggests reflecting on professional practices in general in early childhood, and speech therapy practices in particular. Today, there is no doubt that context-focused speech therapy intervention is defined as a construct and needs not only a reconceptualization, but also broad research that delimits assessment and intervention methodologies within a framework based on research evidence.

Some limitations in the present study should be noted here. First, the sample size. It would be interesting to expand this in order to achieve a better representation of the various autonomous communities of Spain, since these regions have specific socio-cultural characteristics. The expansion of the sample would also allow a greater representation of parents with innatist and environmentalist conceptions.

Also, it would be of interest to have assessments through direct execution methods in parallel, so as to be able to clarify whether the scores obtained in the parental assessment of pragmatic development coincide with those obtained in direct execution tests, as is the case in other studies carried out with the TPP(e) (Botana, 2021).

Another issue that emerges from the present research, and which suggests future lines of work, is the study of the communicative formats of the family and their relationship with pragmatic development. The socio-family variables that indicate better achievement in pragmatic development are traditionally associated with better alternatives for a good work-life balance, and greater access to resources and experiences. This may allow for more time to be dedicated to children, but how this time materializes and to what extent it favors communicative formats that themselves favor pragmatic development is an interesting field of study, one that would allow new strategies in the framework of family-centered speech therapy intervention. A recent study by Martinot et al. (2021) presents a similar proposal regarding exposure to screens in childhood. These authors have shown that screen exposure times are not as much predictors of worse outcomes in children's communicative development as is traditionally believed, but that it is the social moment at which screen exposure occurs that poses more negative effects on communicative development. For example, the children with the worst communicative development skills are those whose families make use of screens during dinner, depriving children of the communicative and social routine that family meals imply, demonstrating the impact of a social routine, in this case dinner, on communicative development.

## 5 Conclusions

The effect of parental conceptions and other socio-family variables on the assessment of pragmatics points to the need to obtain convergent measures in an area as complex as that of communicative development in early childhood, especially considering that a neutral, context-free assessment is not possible, or desirable (Gibbs and Colston, 2020). Pragmatic development must be assessed within this contextual framework, and should take into account each of the variables present therein. Hence the complementarity between parental reports and direct execution tests.

Parental reports have been shown to be a valuable tool in the assessment of children's language development (Šmit Brleković and Kuvač Kraljević, 2023), so much so that their use is common in clinical and scientific settings. The fact that there is a great deal of congruence between educational and socioeconomic level, parental experience, beliefs about the determinants of development, and expectations about the evolutionary calendar and pragmatic development as assessed through the TPP(e), only reinforces the idea that parental reports are a robust indicator of children's development in the family environment.

However, it is not enough to claim that through parental reports it is possible to assess the development of children. It is also useful to explore the explanation of differences between clinical and parental assessments, as these reports may contain assessment biases. But it must also be understood that the assessment of the actions through which a child demonstrates their communicative skills needs be carried out in parallel with the child and their context (Mikulic et al., 2007), thus achieving complementary versions of the same assessment.

In any case, verifying the congruence and interest of the variables studied here for the evaluation of early pragmatic development should not obscure the importance of developing programs aimed at modifying these. In particular, parental belief systems, as well as the information and training that exist and arise within families, are dynamic and modifiable. Family intervention programs, and the incorporation of the social context into the clinical or educational treatment of communication and language problems, are increasingly necessary in today's complex society (Bronfenbrenner, 1986; Bronfenbrenner and Morris, 2006).

In short, such studies, as well as out own here, corroborate the notion that child development is not an isolated activity, but occurs in the context of interactions with caregivers, whose expectations and thinking have a decisive influence on the way they approach their children's development and education.

## Data availability statement

The original contributions presented in the study are included in the article/supplementary material, further inquiries can be directed to the corresponding author.

## Author contributions

IB: Conceptualization, Formal analysis, Funding acquisition, Investigation, Methodology, Project administration, Validation, Visualization, Writing—original draft, Writing—review & editing. MP: Formal analysis, Supervision, Validation, Writing—review & editing.
